# Organic mulch can suppress litchi downy blight through modification of soil microbial community structure and functional potentials

**DOI:** 10.1186/s12866-022-02492-3

**Published:** 2022-06-11

**Authors:** Dandan Xu, Jinfeng Ling, Fang Qiao, Pinggen Xi, Yani Zeng, Jianfan Zhang, Cuizhen Lan, Zide Jiang, Aitian Peng, Pingdong Li

**Affiliations:** 1 Department of Applied Chemistry and Biotechnology/Postdoctoral Innovation Practice Base, Shenzhen Polytechnic, Shenzhen, 518055 China; 2 Department of Plant Protection/Guangdong Province Key Laboratory of Microbial Signals and Disease Control, South China Agricultural University, Guangzhou, 510642 China; 3 Plant Protection Research Institute, Key Laboratory of High Technology for Plant Protection of Guangdong Province, Guangdong Academy of Agricultural Sciences, Guangzhou, 510640 China; 4 Shenzhen Nanshan Xili Orchard, Shenzhen, 518055 China; 5 Shenzhen Agricultural Technology Promotion Center, Shenzhen, 518040 China

**Keywords:** Organic mulch, Litchi plantation, Bacterial and fungal communities, Antimicrobial, Litchi downy blight

## Abstract

**Background:**

Organic mulch is an important management practice in agricultural production to improve soil quality, control crop pests and diseases and increase the biodiversity of soil microecosystem. However, the information about soil microbial diversity and composition in litchi plantation response to organic mulch and its attribution to litchi downy blight severity was limited. This study aimed to investigate the effect of organic mulch on litchi downy blight, and evaluate the biodiversity and antimicrobial potential of soil microbial community of litchi plantation soils under organic mulch.

**Results:**

Organic mulch could significantly suppress the disease incidence in the litchi plantation, and with a reduction of 37.74% to 85.66%. As a result of high-throughput 16S rRNA and ITS rDNA gene illumine sequencing, significantly higher bacterial and fungal community diversity indexes were found in organic mulch soils, the relative abundance of norank f norank o Vicinamibacterales, norank f Vicinamibacteraceae, norank f Xanthobacteraceae, Unclassified c sordariomycetes, *Aspergillus* and *Thermomyces* were significant more than that in control soils. Isolation and analysis of antagonistic microorganism showed that 29 antagonistic bacteria strains and 37 antagonistic fungi strains were unique for mulching soils.

**Conclusions:**

Thus, we believe that organic mulch has a positive regulatory effect on the litchi downy blight and the soil microbial communities, and so, is more suitable for litchi plantation.

## Background

Litchi (*Litchi chinensis* Sonn.), a tropical and subtropical fruit species, is one of the most popular and consumed fruit in the world. From 2014, the planting area, production and output value of litchi in China have ranked first in the world [1]. Litchi crops are subject to downy blight during blooming and fruiting stages, which leads to great economic losses [2]. *Peronophythora litchii*, the pathogen of litchi downy blight, produces sporangia which can germinate or release zoospores for the infection on the host. In addition, the production of oospores existed in soil is responsible for a resultant infection and environmental stresses [3]. 

Soil microorganism consisted of large communities, such as endophytes, symbionts, pathogens, and plant growth promoting rhizobacteria. Soil microbial communities play a critical role in soil quality and ecosystem stability and sustainability [4]. Along with improving soil health and increasing plant’s responses to abiotic stress by altering defense and metabolic pathways, the soil microbiota also provides an important role in suppressing plant disease [5, 6]. Various studies showed that the obvious difference in soil bacterial communities between disease-suppressive soils and disease-conducive soils [7, 8]. Zhang et al. indicated that the decrease of soil microbial diversity was responsible for the development of soil-borne bacterial wilt diseases of tomato [9], which was consistent with the research of Kwak et al. that the high functional redundancy in soil microbial diversity enables wilt resistance in tomato [10].

Currently, some agricultural practices could alter soil environmental factors, and thus, further affected the composition of soil microbial communities. As one important management practice in agricultural production, organic mulch is mainly used for soil improvement and environmental protection. The application of mulch derived from plant residues could not only increase water infiltration [11], prevent soil nutrient loss [12] and suppress weed germination [13], but also control crop pests and diseases [14, 15] and increase the biodiversity of soil microecosystem [16]. Previous studies have reported strong changes in richness and diversity of soil microbial community regulated by organic mulch in vegetable and tea plantation [17, 18]. Organic mulch has been reported to involved with improvement of soil physicochemical properties in litchi orchard [19]. However, to our knowledge, the information about soil microbial diversity and composition in litchi plantation with organic mulch and its effect on litchi downy blight was limited.

In the present study, one field trial was performed to investigate the litchi downy blight under different management methods. Additionally, based on 454-pyrosequencing of the fungal internal transcribed spacer (ITS) region and the bacterial 16S rRNA gene, a comparative microbiome analysis of soils was investigated in the same litchi plantation treated with/without organic mulch. Moreover, the relationship between soil bacterial, fungal community structure and disease-suppression were discussed. This study not only explore the efficacy of organic mulch on the control of litchi downy blight, but also provided theoretical support for the application of organic mulch in litchi plantation.

## Results

### Organic mulch could suppress litchi downy blight

**Fig. 1:**
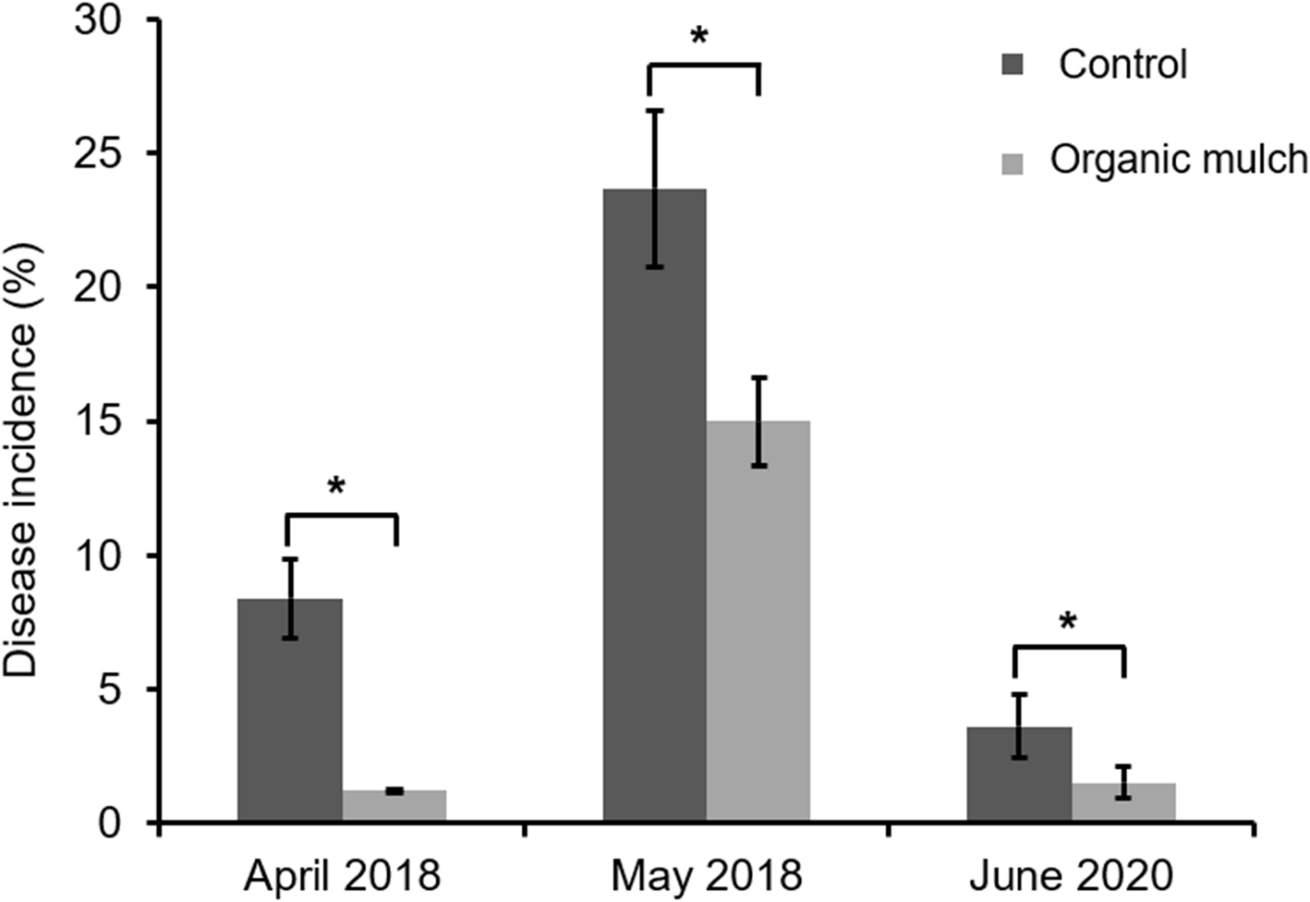
Disease incidence of litchi downy blight from control group and mulch group in 2018 and 2020. For 2018, disease incidence of dropped fruit was investigated on April and May. For 2020, disease incidence of fruit on the tree in different group was conducted on June. Results are represented as the mean of all replicates ± standard error (SE). The asterisks (*) indicate significant difference (*P* ≤ 0.05) between different treatments

To explore the effect of organic mulch on the inhibition of litchi downy blight, disease incidence of litchi planation under organic mulch or conventional tillage methods were investigated. The results showed a significant decrease in the disease incidence of litchi downy blight after the application of organic mulch (Fig. 1). In the investigation on April and May of 2018, the disease incidence of dropped fruits was significantly lower in mulch group than that of in control group, which presented the reducing value of 7.19% and 8.70%, respectively. For the disease investigation on the fruit on the tree, the disease incidence in the control field (3.61%) was significantly higher than that in the organic mulch field (1.51%). Our results inferred that organic mulch could delayed the development of litchi downy blight, which maybe attributed the modification of soil microbial community.

### Changes in the diversity of soil bacterial and fungal community

**Fig. 2:**
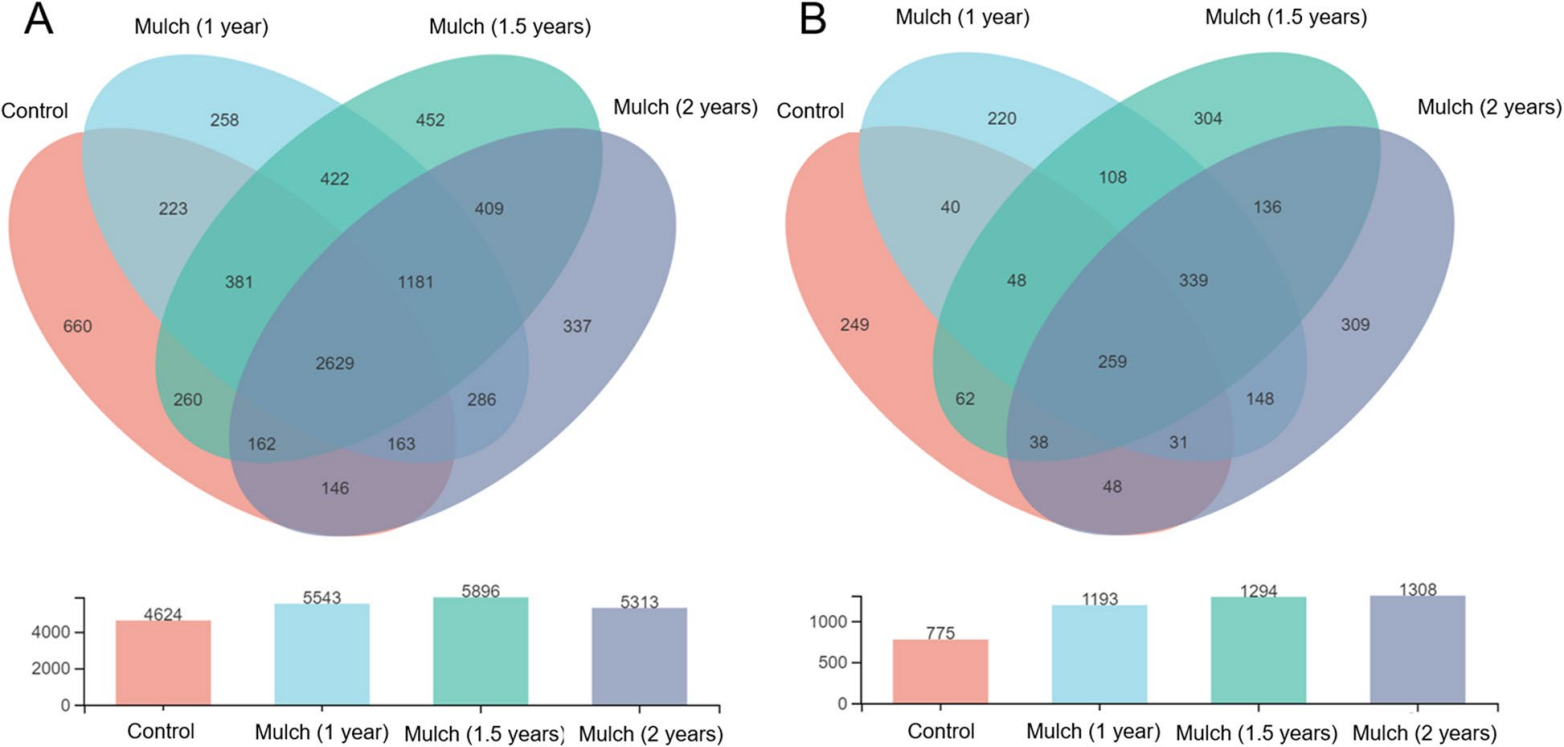
The Venn diagram of microbial communities in soils under different treatments. **A** The number of bacterial OTUs in soils under different treatments; **B** The number of fungal OTUs in soils under different treatments. Control group, bare soil in conventional tillage methods; organic mulch group, soils treated with organic mulch for 1 year, 1.5 years and 2 years

In order to determine the response of soil microorganism to organic mulch, the bacterial and fungal diversity of soil samples treated with/without organic mulch were assessed using phylotype taxonomy. A total of 627,647 high-quality reads of bacteria and 740,047 high-quality reads of fungi were remained in the dataset with the average length of 417 bp and 244 bp, respectively. Through clustering operations, the optimized sequences were classified into operational taxonomic units (OTUs) according to their similarity. With a 3% dissimilarity threshold, the sequences were classified into 8632 and 2175 OTUs in bacterial and fungal communities using the Ribosomal Database Project (RDP) classifier. The Venn diagrams showed that mulching soils under different period (mulch for 1 year, 1.5 years and 2 years) exhibited a greater number of OTUs than control soils (Fig. 2). In bacterial communities, the numbers of OTUs in mulch group for 1 year, 1.5 years and 2 years were more than that in control group, and that in mulch group for 1.5 years was the highest (Fig. 2A). In fungal communities, the numbers of OTUs in three mulching treatmens (mulch for 1 year, 1.5 years and 2 years) were significantly greater than that in control group, and the highest number of fungal OTUs was 1308 which detected in mulch group for 2 years (Fig. 2B).

To quantify the diversity and richness of microbial community of soils among different treatments, the microbial community α-diversity were evaluated by the Ace, Chao1, Shannon and Simpson within a single microbial ecosystem, is shown in Table 1. The coverage indexes from 24 soil samples were greater than 0.97, showed that the sequencing capacity were acceptable. The richness (Ace and Chao1 indices) and diversity (Shannon and Simpson) of bacteria and fungi calculated based on the rarefied sequences showed that mulch treatment and different mulch period affected the bacterial and fungal communities. In bacterial communities, the richness (Ace and Chao1 indices) and Shannon diversity of mulch group for 2 years was higher than that in control group and mulch group for 1 year and 1.5 years; while in fungal communities, the richness (Ace and Chao1 indices) of control group was higher that in mulch soils (mulch for 1 year, 1.5 years and 2 years). However, the Simpson diversity of control group in bacterial community was slight higher than that in mulch soils (mulch for 1 year, 1.5 years and 2 years), while in fungal community, the Simpson diversity of control group was significantly lower than that in mulch soils (mulch for 1 year, 1.5 years and 2 years).

**Table 1:** The diversity and richness indices of soil bacterial and fungal communitites

	**Control**	**Mulch (1 year)**	**Mulch (1.5 years)**	**Mulch (2 years)**
**Bacteria**				
Ace	3965.47 ± 60.20a	4920.88 ± 66.58b	5004.96 ± 61.42c	5388.96 ± 56.98d
Chao1	3889.78 ± 37.22a	4863.81 ± 50.06b	5025.71 ± 11.38c	5086.18 ± 44.44d
Shannon	6.67 ± 0.05a	6.98 ± 0.03b	6.95 ± 0.01b	7.05 ± 0.06b
Simpson (%)	0.33 ± 0.01b	0.22 ± 0.01a	0.25 ± 0.01a	0.22 ± 0.01a
Coverage (%)	97.65 ± 0.14a	97.12 ± 0.11a	97.16 ± 0.45a	97.07 ± 0.09a
**Fungi**				
Ace	983.32 ± 19.59b	764.41 ± 33.17a	732.07 ± 35.70a	888.89 ± 9.81b
Chao1	989.24 ± 17.79c	778.13 ± 51.10ab	748.22 ± 38.41a	886.31 ± 10.85bc
Shannon	4.63 ± 0.16b	3.25 ± 0.39a	4.11 ± 0.20ab	3.97 ± 0.24ab
Simpson (%)	2.42 ± 0.51a	16.49 ± 2.09b	4.67 ± 0.76a	5.47 ± 0.30a
Coverage (%)	99.81 ± 0.03a	99.69 ± 0.02a	99.81 ± 0.06a	99.77 ± 0.04a

Control group, bare soil in conventional tillage methods; organic mulch group, soils treated with organic mulch for 1 year, 1.5 years and 2 years. Data are presented as mean values ± SE (Standard error). Different letters indicate significant difference among different treatment, according to statistics analysis using SPSS statistics 23 with Duncan’s multiple range test (*P* ≤ 0.05)

**Fig. 3:**
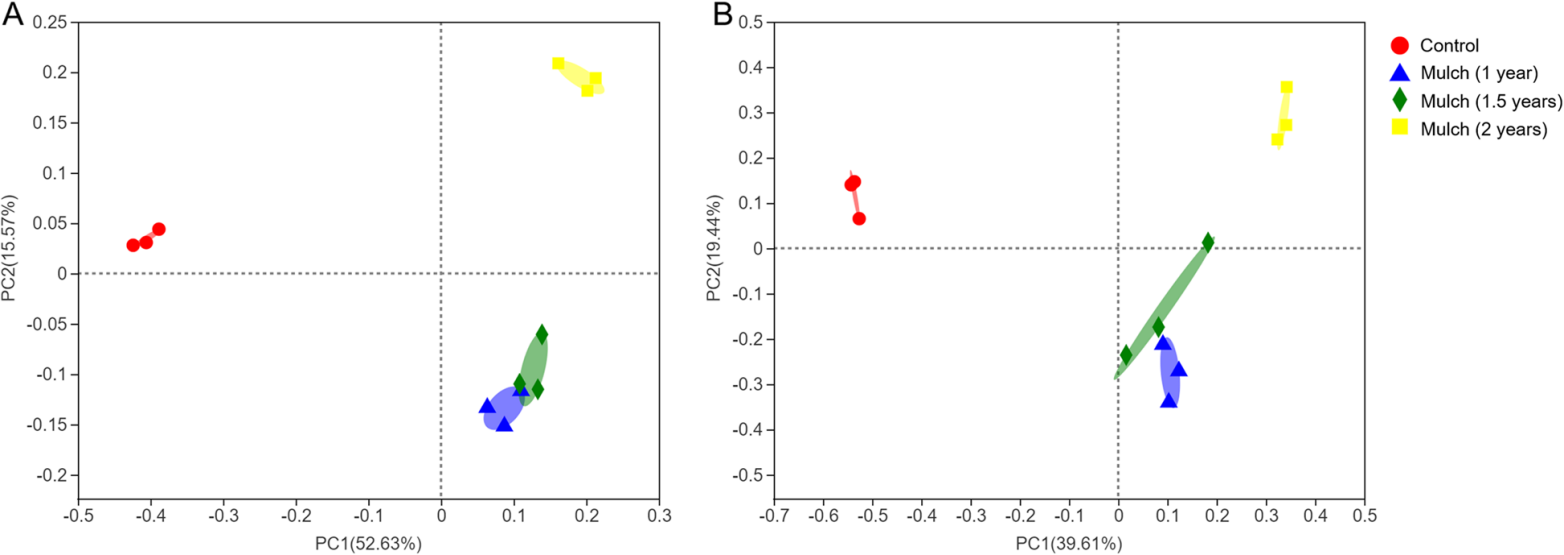
The principal co-ordinates analysis (pCoA) based on the Bray–Curtis distance in microbial communities in soils under different treatments. **A** The distribution of bacterial communities in soils under different treatments; **B** The distribution of fungal communities in soils under different treatments. Control group, bare soil in conventional tillage methods; organic mulch group, soils treated with organic mulch for 1 year, 1.5 years and 2 years

To get a better insight into the differences of the soil microbial communities, the principal coordinates analysis (PCoA) based on the Bray–Curtis distance was applied to evaluate the microbial community β-diversity. As shown in Fig. 3A, samples of control group and mulch group for 2 years were distributed separately at 52.63% and 15.57% on the pCoA vector x and y axes for the bacterial community, while mulch group (1.5 years and 2 years) were contiguous but distinct from control group and mulch group for 2 years. Likewise, the pCoA variation (39.61% for PC1 and 19.44% for PC2) accounted for the fungal community across all samples (Fig. 3B). All soil samples distinct from the others expect for mulch group (1 year and 1.5 years), demonstrated that large microbial community differences affected by organic mulch and different mulch period.

### Bacterial communities in the Soil

**Fig. 4:**
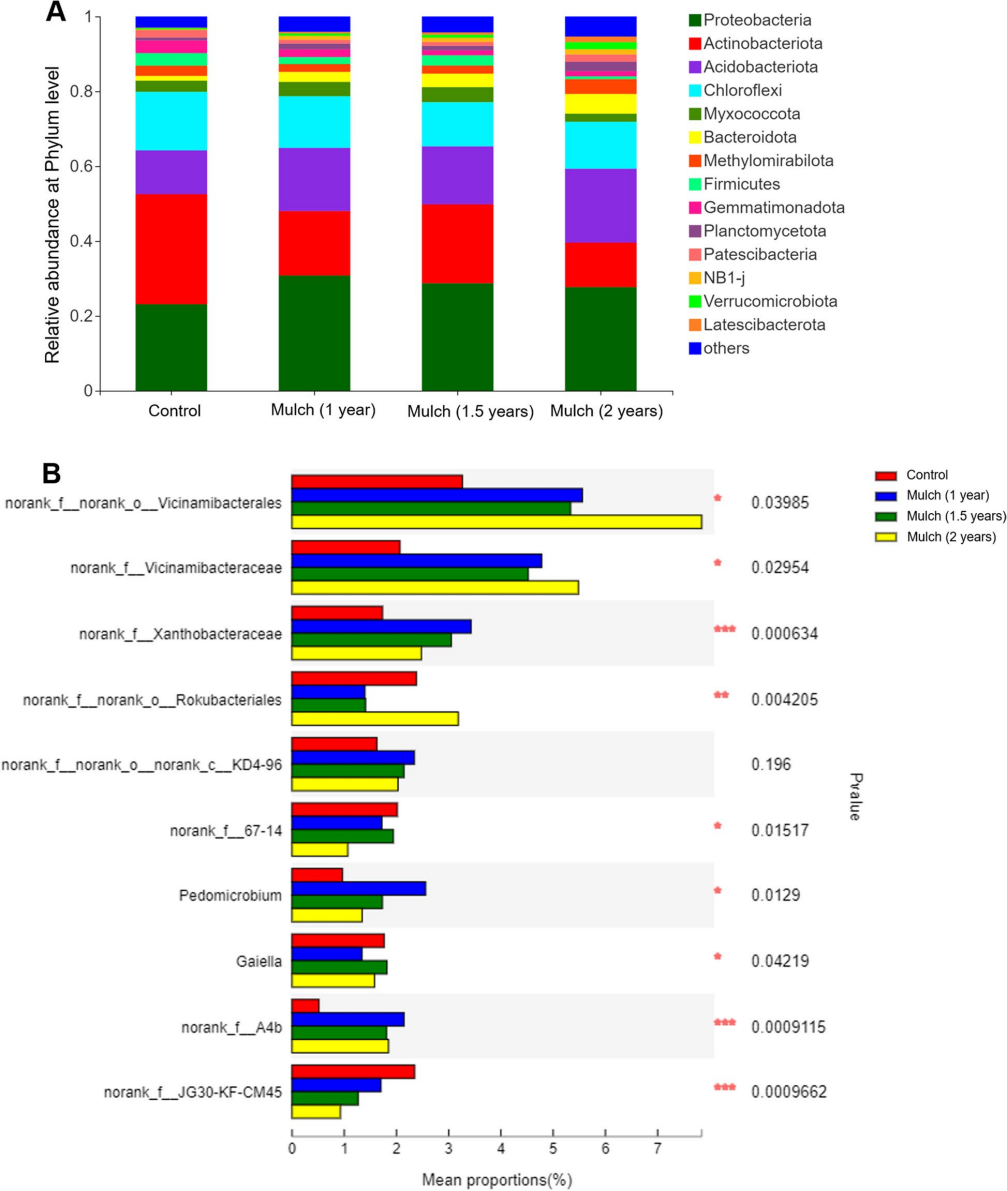
The relative abundance of main bacterial communities in soil under different treatments. **A** Relative abundance of the dominant bacterial community at phylum level; **B** Relative abundance of top 10 bacterial community at genus level. The “others” comprise the unclassified and low-abundance phyla. Control group, bare soil in conventional tillage methods; organic mulch group, soils treated with organic mulch for 1 year, 1.5 years and 2 years. The Scheffe’s value cutoff was 0.95, ***p ≤ 0.001, **0.001 < *p* ≤ 0.01, and *0.01 < *p* ≤ 0.05

Obvious differences in the composition and diversity of the bacterial communities were found across the soil. As shown in Fig. 4A, Proteobacteria, Actinobacteria, Acidobacteria and Chloroflexi, in rank order, were the abundant phyla in both treatments, and Proteobacteria, Acidobacteria and Bacteroidota in mulch soils (mulch for 1 year, 1.5 years and 2 years) were significantly larger than in control soil. At the genus level, relative abundance of top 10 bacterial communities was identified, and top 3 microbial communities (norank_f_norank_o_Vicinamibacterales, Norank_f__Vicinamibacteraceae, Norank_f__Xanthobacteraceae) significantly increased in response to organic mulch (Fig. 4B).

### Fungal communities in the soil

**Fig. 5:**
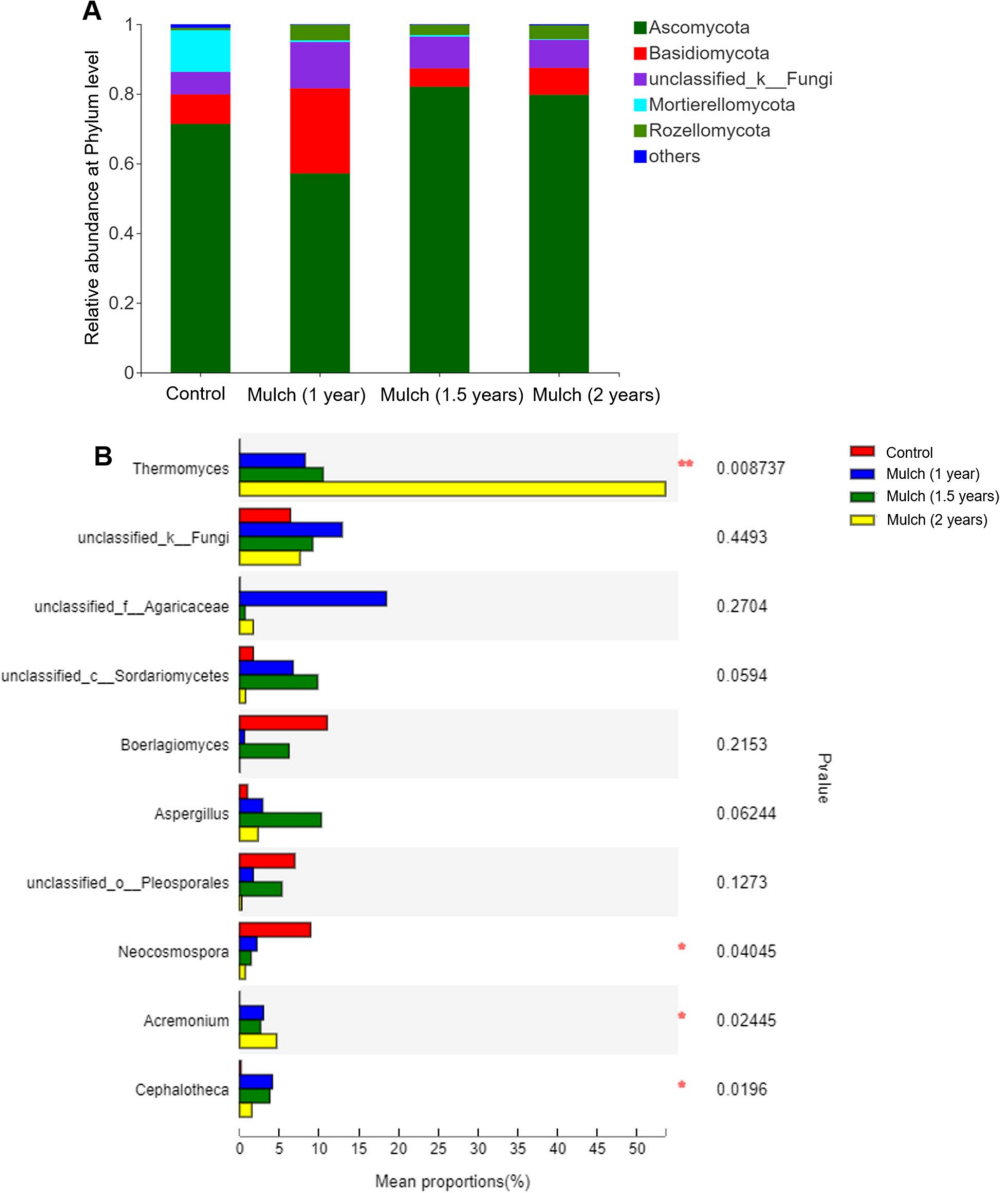
The relative abundance of main fungal communities in soil under different treatments. **A** Relative abundance of the dominant fungal community at phylum level; **B** Relative abundance of top 10 fungal community at genus level. The “others” comprise the unclassified and low-abundance phyla. Control group, bare soil in conventional tillage methods; organic mulch group, soils treated with organic mulch for 1 year, 1.5 years and 2 years. The Scheffe’s value cutoff was 0.95, ***p ≤ 0.001, **0.001 < *p* ≤ 0.01, and *0.01 < *p* ≤ 0.05

To dissect the taxonomic composition of fungal communities in the soil, relative abundance of the dominant fungal communities at phylum and genus levels were aligned. At the phylum level, Ascomycota was significantly dominated across both treatments, and its relative abundance in mulch group (1.5 years and 2 years) significantly higher compared to control soil (Fig. 5A). Notably, organic mulch markely increased the relative abundance of *Thermomyces, Aspergillus* and *Acremonium* in the soil, whereas, the relative abundance of *Neocosmospora* in fungal community were significantly decreased with the increasing period of mulch treatment (Fig. 5B).

### Identification of antagonistic bacteria contributing to antimicrobial activity

**Fig. 6:**
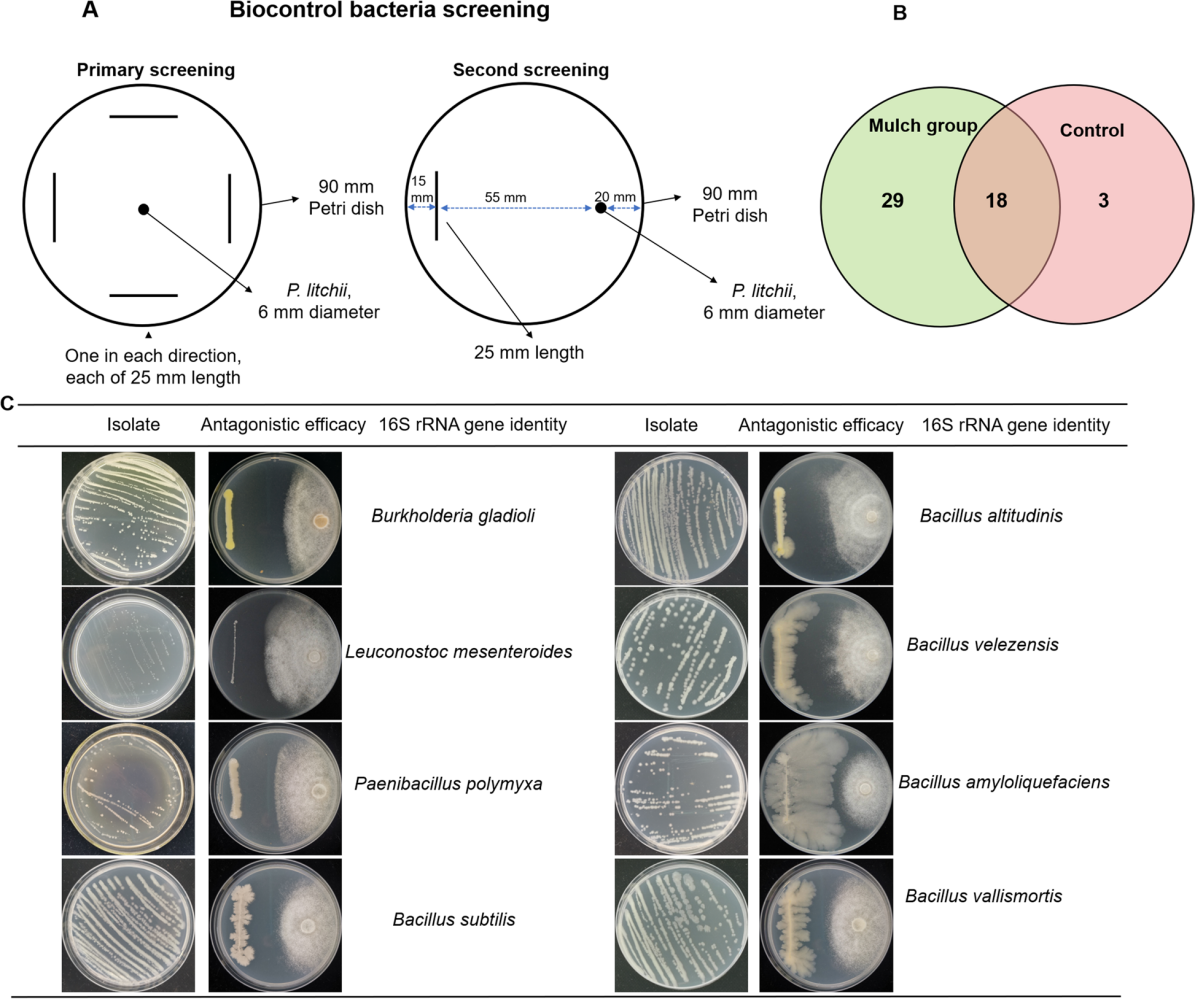
Screening and characterization of antagonistic bacteria isolates detected in this study. **A**, The method of biocontrol bacteria screening; **B** Venn diagram for common and unique strains detected from mulch soils (mulch for 2 years) and control soils (bare soil in conventional tillage methods); **C** Characterization of antagonistic bacteria isolates detected in this study

The richness and species of antagonistic bacteria were positively associated with the survival of pathogen. From the primary and second screening, a total of 50 antagonistic bacteria strains were obtained, with 18 strains were common in both treatments, and 29 and 3 strains were unique for mulch soils and control soils, respectively (Fig. 6B). Based on the sequence similarities to the 16S rRNA gene sequences, 8 different species of antagonistic bacteria with excellent antimicrobial efficacy were identified. These were *Burkholderia gladioli*, *Leuconostoc mesenteroides*, *Paenibacillus polymyxa*, *Bacillus subtilis*, *B. altitudinis*, *B. velezensis*, *B. amyloliquefaciens*, and *B. vallismortis* (Fig. 6C).

### Identification of antagonistic fungi contributing to antimicrobial activity

**Fig. 7:**
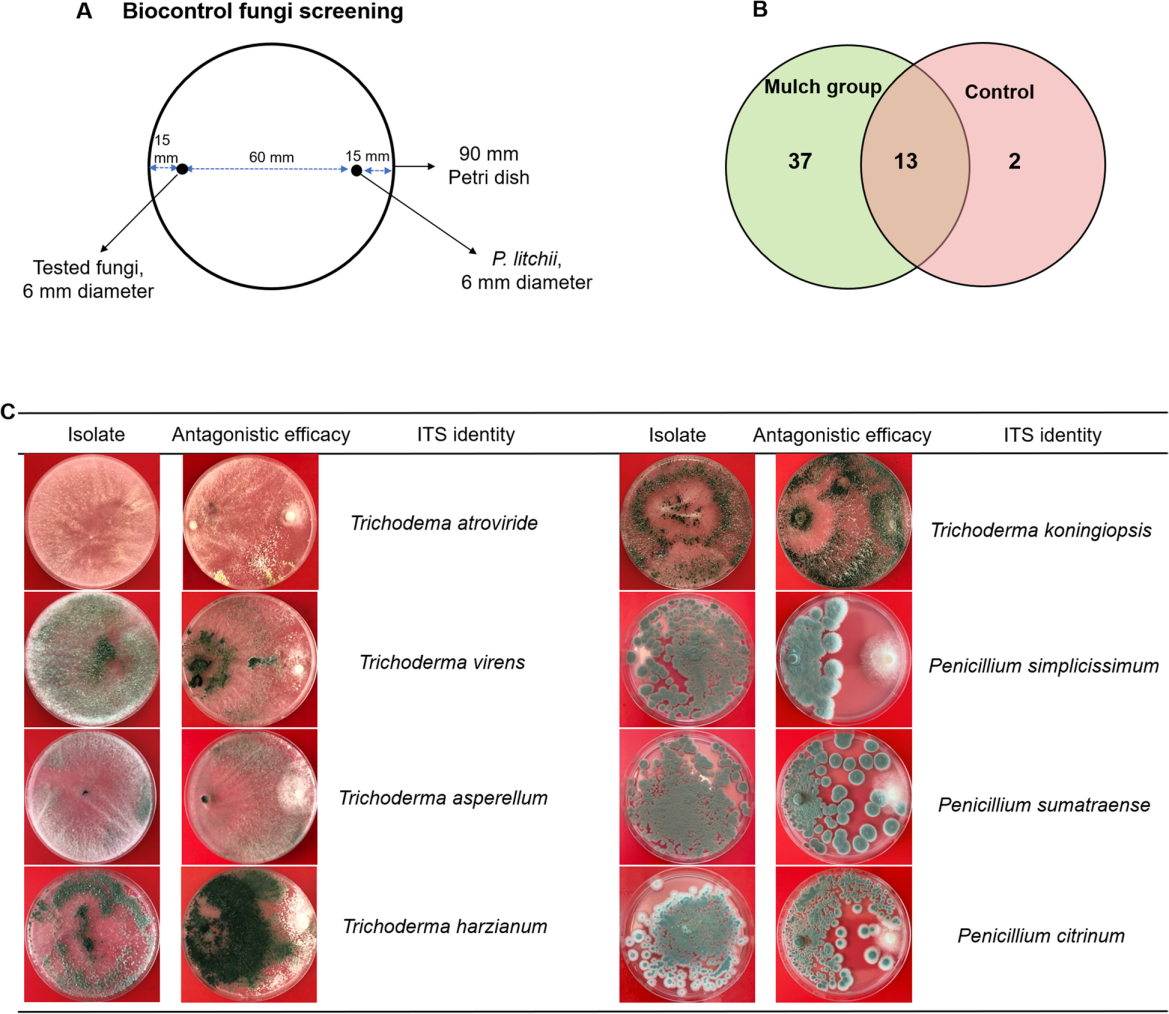
Screening and characterization of antagonistic fungi isolates detected in this study. **A** The method of biocontrol fungi screening; **B** Venn diagram for common and unique strains detected from mulch soils (mulch for 2 years) and control soils (bare soil in conventional tillage methods); **C** Characterization of antagonistic fungi isolates detected in this study

To evaluate the antimicrobial activity of enrichment fungal cultures against *P. litchii*, primary and second screening test were conducted. In total, 52 antagonistic fungal strains were obtained, with 13 strains were common in both treatments, and 37 and 2 strains were unique for mulch soils and control soils, respectively (Fig. 7B). As a result of ITS sequences alignment, eight major species of antagonistic fungi with excellent antimicrobial efficacy were identified from organic mulch soils, which belonging to *Trichoderma* sp. and *Penicillium* sp. (Fig. 7C).

## Discussion

Organic mulch was widely adopted in agricultural cultivation for a long time. The general impact of mulch on economy and environment have been fully recognized [20]. For example, some organic mulches could act as sponge and retain rainfall thus providing water for crop requirement, and an 43% reduction of runoff was obtained via the application of orgainc mulch [21]. Mulches also could potentially minimize nutrient losses and to enhance crop yield [22]. Additionally, mulch provides potential advantages in agro-ecological systems, which could improve the recycling of organic waste, remediate the heavy metals, and diminish the pesticide use [23]. In terms of the influence of organic mulch on the control of disease, some research gave the results that pathogens insidde the mulch materials will transfer to healthy plants and increase the chances of disease occurrence [24]. Whereas, our study indicate that organic mulch application led to a significant decline in the disease incidence of litchi downy blight, which in consistent with research of Tymon et al. that mulches combined with endophytic fungi could lessen the disease caused by *Verticillium dahliae* [25].

Mulches possess the ability of disease reduction in crops mainly through a direct or an indirect mechanism. Mulches could markedly reduce the evaporation losses and maintain the soil mositure, hence directly act as barries against irrigation water or beating action of rain drops which might carrry conidia of different pathogen [26]. The indirect effects of mulches are helpful for the diminution of diseases mainly via providing nutrition for many beneficial organisms which competes the ecological niches or release the chemicals for the inhibition of pathogens, thus reducing the chances of disease occurrence [27]. Consequently, mulches provide a healthy atmosphere for the growth and development of crop plants and hence act as barriers against pathogenic organisms.

Soil microbiota is considered to be a critical factor to regulate soil quality and sustainability, and its community and diversity are significant involved with soil–plant health via triggering different functional roles, which including decomposing organic matter, ecosystem regulators, and biological antagonism [28–30]. Our present study indicated that organic mulch increase the community diversity and populations of beneficial microorganisms, which provide an explanation for the reduction of litchi downy blight induced by mulch application.

Soil microbiota are closely related to soil quality and ecosystem stability and sustainability, which are crucial for plant health and productivity [31]. Various studies demonstrated the alteration of soil microbial community was closely associated with soil suppressiveness to soilborne pathogenic fungi [32, 33]. Wu et al. highlighted the close association between replant disease and the variations in structure and potential functions of rhizosphere bacterial community [4]. Yang et al. indicated that soil microbial diversity had a strong effect on tobacco wilt disease level [34]. In addition, any modifications in soil microbial community assemblages will have a cascade of effects on soil structure and nutrient cycling, including soil aggregate stability and decomposition processes [35, 36]. The results of biodiversity measurement showed that the phyla Proteobacteria, Actinobacteria, Acidobacteria and Chloroflexi were dominant bacteria, which are consistently predominant bacteria in tillage soil [37]. Proteobacteria plays an important role in straw decomposition and soil nutrient uptake [38], and Acidobacteria is considered to have extensive metabolic and genetic functions [39]. Therefore, the increase pf relative abundance of phyla Proteobacteria and Acidobacteria under organic mulch application might be attribute to organic matter incorporation. The fungal taxonomic composition and richness of functional fungi in the soils under organic mulch methods also significantly higher than that in bare soils with conventional tillage methods. The dominant *Aspergillus* and *Thermomyces* genera were observed under organic mulch application. Certain affiliated genera including *Thermomyces* spp. are the key contributors to the hemicellulose hydrolysis during root-surrounding decomposition, which supply nutrients to microbial system in the soils [40].

## Conclusions

Litchi downy blight caused by *P. litchii* is one of the most destructive diseases in litchi planation. Our findings revealed that reduction of litchi downy blight induced by organic mulch was closely associated with dysbiosis of soil microbiota. The organic mulch application could delay this disease mainly depend on reshaping the soil microbial community and modifying the potential functions microbes that harbor antagonistic activities against *P. litchii* and contribute to soil suppressiveness. Our results reinforce the influence of organic mulch on disease control and soil microbial diversity in litchi plantation from the aspects of microbial structure and ecological function, which provide a suitable method for litchi plantation. Further work is needed to investigate the survival of causal agent in the presence of beneficial microorganisms and to link the potential functions to organic mulch application.

## Materials and methods

### Field experiments

The field experiments were conducted from 2018 to 2020 at Xili Orchard in Shenzhen of China (22.3°N and 113.5°E). The cultivated litchi is ‘Nuomici’. The field was divided into two blocks and the treatments were: (1) the control group: bare soil in conventional tillage methods, (2) the mulch group: soil covered with litchi shredded branches tilled to a depth of 8–10 cm for 1 year, 1.5 years, and 2 years. To discover disease incidence and shifts in the soil microbiome, same conventional cultivation was carried out in the control group and mulch groups.

### Disease incidence discovery

In 2018 and 2020, disease incidence was investigated by calculating the disease incidence of dropped fruits and the litchi fruit on trees. During April and May of 2018, dropped fruits in the control field and mulch field were collected and put into plastic boxes to keep humidity. After 2–3 d, fruitlets covered with white mold were counted and the disease incidences were examined. Each treatment was conducted by collecting fruits from 5 different trees for one repeat, and at least 200 fruits for each repeat was calculated. In June 2020, 15 trees in the control group and mulch group were randomly selected and each repeat contained 5 trees. Disease incidence of the trees were determined by calculating 30 fruits in each of the four directions (east, south, west and north) of each tree, and diseased fruit with downy white sporangiophores were counted.

### Soil sampling collection

For each treatment, 4 different soil samples from different trees were collected. The soils from 15 cm depth were placed in sterile plastic bags and transported to the laboratory in an icebox immediately. The samples were stored at -80 ℃ until high-throughput sequencing and analysis.

### DNA extraction

Aliquots (0.25 g) of the soil samples were processed using a MOBIO PowerSoil® kit. The extracted DNA samples were analyzed using a NanoDrop 2000 UV–Vis spectrophotometer (Thermo Scientific, Wilmington, DE, USA). The DNA quality was confirmed by 1% agarose gel electrophoresis. The extracted DNA samples were selected and used to conduct microbial community analysis by PCR using primers 338F (5′-ACTCCTACGGGAGGCAGCAG-3′) and 806R (5′-GGACTACHVGGGTWT CTAAT-3′) for 16S rDNA in bacteria [41], and primers ITS1F (5′-CTTGGTCATTTAGAGGAAGTAA-3′) and ITS2R (5′-GCTGCGTTCTTCATCGATGC-3′) for ITS in fungi [42]. The PCR reactions were performed in triplicate, using 20 μL mixtures containing 4 μL 5 × FastPfu buffer, 2 μL 2.5 mM dNTPs, 1 μL primer mix (5 μL), 0.4 μL FastPfu polymerase, and 5 ng extracted DNA as the template. The PCR products were extracted from a 2% agarose gel and further purified using the AxyPrep DNA Gel Extraction Kit (Axygen Biosciences, Union City, CA, USA). The products were quantified using QuantiFluor-ST (Promega, Madison, USA). Purified amplicons were then pooled in equimolar concentrations and paired-end sequenced (2 × 300) using the Illumina MiSeq platform (Illumina, San Diego, CA, USA) according to the standard protocols of Shanghai Majorbio Bio-pharm Technology Co., Ltd. Raw sequences were filtrated using FASTX Toolkit 0.0.12 software to remove low quality reads with Q value < 20 and less than 35 bp.

### Illumina sequencing and processing of sequencing data

The purified amplicons were pooled on the Illumina MiSeq platform (Illumina, San Diego, USA) of equal molecular weight and paired-end sequencing (2 × 300) according to the standard protocol of MajorbioBio-Pharm Technology Co. Ltd. (Shanghai, China). The original sequencing sequence was controlled using Trimmomatic software and merged by FLASH software. The specific criteria are consistent with previous study [43]. UPARSE (version 7.1; http://drive5.com/uparse/) was used to cut the similarity of the operational classification units (OTUs) to 97%. http://drive5.com/uparse/) uses a novel “greedy” algorithm that could perform chimera filtering and OTU clustering at the same time. Using the confidence threshold of 70%, the classification of each 16S rRNA gene sequence was analyzed against the Silva database (Release132; http://www.arb-silva.de) through the RDP classifier algorithm (http://rdp.cme.msu.edu/). Using the confidence threshold of 70%, the classification of each ITS sequence was analyzed against the Unite database (version 7.2; http://unite.ut.ee/index.php) through the RDP classifier algorithm.

### Selection and identification of biocontrol bacteria and fungi

Soils from litchi plantation treated with organic mulch for 2 years or without organic mulch were collected and used for bacteria and fungi isolation. Serial dilution method was used to isolate bacteria by dissolving soil samples (25 g) with sterile water (100 mL) in a 250 mL sterilized conical flask and shaking for 30 min using a rotary shaker at 150 rpm, then the resulting solutions were serially diluted up to 10^–3^, spread on LB plates and incubated at 30 ℃ for 24 h for bacteria inoculation, and incubated on PDA plates for 3 d at 25 ℃ for fungi inoculation [44]. Singal colonies were transferred to new LB plates (for bacteria) or PDA plates (for fungi) and used for antagonistic microorganism selection.

The antagonistic abilities of tested bacteria isolates were determined by primary and second screening as shown in Fig. 6A, and the potential antagonistic activity of fungi isolates were measured as shown in Fig. 7A. After incubating on PDA plates for 6 d, the diameters of the pathogen zone of mycelium growth inhibition around bacteria were measured and recorded.

Then, the bacteria or fungi with markedly inhibitory activity were selected and identified by amplifying 16S rRNA gene sequence of tested bacteria using universal primers 27F and 1492R, and amplifying internal transcribed spacer (ITS) of tested fungi using universal primers ITS1 and ITS4 [45, 46]. The obtained PCR products were sequenced and the sequences were subjected to Basic Local Alignment Search Tool (BLAST) searches using the National Center for Biotechnology Information (NCBI) database (http://www.ncbi.nlm.nih.gov) to compare with other sequences deposited in GenBank.

## Data Availability

Raw data for bacterial 16S rRNA and fungal ITS sequence were depositied in the NCBI Sequence Read Archive (SRA) database under accession number PRJNA776979 and PRJNA776976, respectively.
